# Prevalence and genetic characteristics of *Salmonella* in free-living birds in Poland

**DOI:** 10.1186/s12917-015-0332-x

**Published:** 2015-01-31

**Authors:** Marta Krawiec, Maciej Kuczkowski, Andrzej Grzegorz Kruszewicz, Alina Wieliczko

**Affiliations:** Department of Epizootiology and Clinic of Bird and Exotic Animals, Faculty of Veterinary Medicine, Wrocław University of Environmental and Life Sciences, Pl. Grunwaldzki 45, 50-366 Wrocław, Poland; Warsaw Zoological Garden, ul. Ratuszowa 1/3, 03-461 Warsaw, Poland

**Keywords:** Free-living birds, *Salmonella* spp, Poland, Virulence genes, ERIC-PCR

## Abstract

**Background:**

*Salmonella* species are widespread in the environment, and occur in cattle, pigs, and birds, including poultry and free-living birds. In this study, we determined the occurrence of *Salmonella* in different wild bird species in Poland, focusing on five *Salmonella* serovars monitored in poultry by the European Union: *Salmonella* serovars Enteritidis, Typhimurium, Infantis, Virchow, and Hadar. We characterized their phenotypic and genetic variations.

Isolates were classified into species and subspecies of the genus *Salmonella* with a polymerase chain reaction (PCR) assay. The prevalence of selected virulence genes (*spv*B, *spi*A, *pag*C, *cdt*B, *msg*A, *inv*A, *sip*B, *prg*A, *spa*N, *org*A, *tol*C, *iron*N, *sit*C, *ipf*C, *sif*A, *sop*B, and *pef*A) among the isolated strains was determined. We categorized all the *Salmonella* ser. Typhimurium strains with enterobacterial repetitive intergenic consensus (ERIC)-PCR.

**Results:**

Sixty-four *Salmonella* isolates were collected from 235 cloacal swabs, 699 fecal samples, and 66 tissue samples (6.4% of 1000 samples) taken from 40 different species of wild birds in Poland between September 2011 and August 2013. The largest numbers of isolates were collected from Eurasian siskin and greenfinch: 33.3% positive samples for both. The collected strains belonged to one of three *Salmonella* subspecies: *enterica* (81.25%), *salamae* (17.19%), or *houtenae* (1.56%). Eighteen strains belonged to *Salmonella* ser. Typhimurium (28.13%), one to ser. Infantis (1.56%), one to ser. Virchow (1.56%), and one to ser. Hadar (1.56%). All isolates contained *spiA*, *msg*A, *inv*A, *lpf*C, and *sif*A genes; 94.45% of isolates also contained *sit*C and *sop*B genes. None of the *Salmonella* ser. Typhimurium strains contained the *cdtB* gene. The one *Salmonella* ser. Hadar strain contained all the tested genes, except *spv*B and *pef*A; the one *Salmonella* ser. Infantis strain contained all the tested genes, except *tspv*B, *pef*A, and *cdtB*; and the one *Salmonella* ser. Virchow strain contained all the tested genes, except *spv*B, *pef*A, *cdtB*, and *tolC*.

The *Salmonella* ser. Typhimurium strains varied across the same host species, but similarity was observed among strains isolated from the same environment (e.g., the same bird feeder or the same lake).

**Conclusions:**

Our results confirm that some wild avian species are reservoirs for *Salmonella* serotypes, especially *Salmonella* ser. Typhimurium.

## Background

*Salmonella* species are widespread in nature, and occur as pathogenic bacteria in the intestines of domestic and wild animals, including birds. Cases of suspected bird-to-human transmission of *Salmonella* have been reported [1]. Most identified *Salmonella* serovars have been *Salmonella enterica* and almost all are able to cause illness in humans and animals [2]. The most frequently reported serotypes causing human salmonellosis in the European Union (EU) are *S. enterica* subsp. *enterica* serovar (ser.) Enteritidis and *S. enterica* subsp. *enterica* ser. Typhimurium [3]. Because of the suspected high correlation between salmonellosis in poultry and the number of human infections, Directive 2003/99/EC of the European Parliament and Council requires that the following five serotypes of *Salmonella* be monitored in poultry flocks: Enteritidis, Typhimurium, Virchow, Hadar, and Infantis. Some strains of *Salmonella* ser. Typhimurium have been identified as host adapted and a cause of salmonellosis in pigeons [4] and passerines [5]. Infections with different serotypes of *Salmonella* have also been documented in gulls, crows, vultures, and parrots [6,7]. *Salmonella* is an environmentally persistent pathogen that can survive and proliferate in diverse environments, including in animals that form part of the human food chain [8]. The molecular characterization of *Salmonella* serovars isolated from poultry, food, and the environment has been reported (e.g., virulence genes and the homology of strains) [9-12]. In contrast, there are few reports of the characterization of strains isolated from wild birds throughout the world. The aim of this study was to isolate and characterize *Salmonella* strains from selected free-living bird species in Poland.

## Methods

During the period from September 2011 to August 2013, 1000 samples were collected: 235 cloacal swabs from four species of aquatic wild birds, and 699 fecal samples and 66 tissue samples from 36 different species of free-living birds (Table 1). Birds found dead and feces were collected by ornithologists from live and dead individuals in six different regions of Poland during the following bird-ringing seasons:

**Table 1:** ***Salmonella*** **isolates obtained from free-living birds**

**No.**	**Origin**	**Type of material**	**Total amount of tested individuals**	**Positive samples** **(%)**	Environmental data */**
1	Mallard duck *Anas platyrhynchos*	cloacal swabs	121 (d)	8 (6,61)	1/ A
2	Great cormorant *Phalacrocorax carbo*	cloacal swab	77 (d)	8 (10,39)	1/A
3	Velvet scoter *Melanitta fusca*	cloacal swab	30 (d)	0 (0,00)	7/A
4	Black coot *Fulica atra*	cloacal swab	7 (d)	0 (0,00)	1/B
5	Mute swan *Cygnus olor*	feces	27 (a)	0 (0,00)	1,2/A
6	Whooper swan *Cygnus cygnus*	feces	6 (a)	0 (0,00)	1,2/A
7	Great tit *Parus major*	feces/tissue	109 (92a/17d)	10 (9,17)	3,4,5,6/B
8	Blue tit *Cyanistes caeruleus*	feces/tissue	43 (36a/7d)	1(2,32)	3,4,5,6/ C
9	Eurasian tree sparrow *Passer montanus*	feces/tissue	53 (48a/5d)	2 (3,77)	3,4,5,6/C
10	Redpoll *Carduelis cabaret*	feces	57 (a)	1(1,75)	6/ A
11	**Eurasian siskin** ***Carduelis spinus***	feces/tissue	**48 (39a/9d)**	**16 (33,3)**	3,4,5,6/ A
12	Common chiffchaff *Phylloscopus collybita*	feces	45 (a)	0 (0,00)	5,6/A
13	Bluethroat *Luscinia svecica*	feces	43 (a)	0 (0,00)	3,4,5,6 /A
14	European robin *Erithacus rubecula*	feces	36 (a)	0 (0,00)	5,6/ A
15	Common reed bunting *Emberiza schoeniclus*	feces	35 (a)	0 (0,00)	3,4,5,6/
16	Eurasian blackcap *Sylvia atricapilla*	feces	35 (a)	0 (0,00)	3,4,5,6/B
17	**Greenfinch** ***Carduelis chloris***	feces/tissue	**30 (20a/10d)**	**10 (33,3)**	3,4,5,6/C some populations A
18	Pied flycatcher *Ficedula hypoleuca*	feces	19 (a)	0 (0,00)	6/ A
19	Hedge sparrow *Prunella modularis*	feces	17 (a)	0 (0,00)	5,6/ B
20	Barn swallow *Hirundo rustica*	feces	17 (a)	0 (0,00)	3,4,5,6/A
21	Common starling *Sturnus vulgaris*	feces/tissue	16 (13a/3d)	3 (18,75)	3,4,5,6
22	Eurasian reed warbler *Acrocephalus scirpaceus*	feces	15 (a)	0 (0,0)	5,6/A
23	Fieldfare *Turdus pilaris*	feces	13(a)	0 (0,0)	5,6/A
24	Yellow wagtail *Motacilla flava*	feces	13 (a)	0 (0,0)	3,4,5,6/ A
25	Blackbird *Turdus melura*	feces/tissue	11 (10a/1d)	1 (9,09)	3,4,5,6/B
26	Common chaffinch *Fringilla coelebs*	feces	9(a)	0 (0,00)	3,4,5,6/B
27	Whitethroat *Sylvia borin*	feces	9 (a)	0 (0,00)	5,6/A
28	Yellow- hammer *Emberiza citrinella*	feces	7 (a)	0 (0,00)	3,4,5,6/B
29	Lesser whitethroat *Sylvia curruca*	feces	7 (a)	0 (0,00)	5,6/A
30	Long-tailed tits *Aegithalos caudatus*	feces	6 (a)	0 (0,00)	6/B
31	Hooded crow *Corvus cornix*	tissue	6 (d)	0 (0,00)	2/B
32	Rook *Corvus frugilegus*	feces/tissue	6 (3a/3d)	1 (16,66)	2/A
33	Common wood pigeon *Columba palumbus*	feces/tissue	6 (2a/4d)	1 (16,67)	2/A
34	Common swift *Apus apus*	feces/tissue	5 (4a/1d)	1 (20,00)	3,4,5,6/A
35	Willow worbler *Phylloscopus trochilus*	feces	5(a)	0 (0,00)	6/A
36	Willow tit *Poecile montanus*	feces	5 (a)	0 (0,00)	3,4,5,6/ B
37	Eurasian marsh harrier *Circus aeruginosus*	feces	1(a)	1 (100,00)	8/A
38	Sparrowhawk *Accipiter nisus*	feces	1(a)	0 (0,00)	8/B
39	Common buzzard *Buteo buteo*	feces	1 (a)	0 (0,00)	9/B
40	Golden eagle *Aquila chrysaetos*	feces	3(a)	0 (0,00)	9/C

d, dead individuals; a, alive individuals;

The boldfaces indicate the species of birds with the highest amount (percent) of positive samples.

*Locations of sample collection:

1. Lakes of the Lower Silesia region (southern Poland).

2. Parks of Wrocław (southern Poland).

3. Bird feeders in Wrocław city center (southern Poland).

4. Bird feeders in the suburbs of Wrocław (southern Poland).

5. Rakutowskie Lake of Kuyavian-Pomeranian Voivodeship (middle Poland).

6. Sudetic Mountains (southern Poland).

7. Baltic coast (northern Poland).

8. Wildlife rescue center in Lower Silesia (southern Poland).

9. Wildlife rescue center in Greater Poland (middle Poland).

**Lifestyles of birds: A, migratory bird; B, partially migratory bird; C, resident.

- winter and early spring in the Wrocław city center, suburbs, and parks, the ponds in the Lower Silesia region, the Baltic coast, and two wildlife rescue centers;
- summer and early autumn in the Rakutowskie Lake of Kuyavian–Pomeranian Voivodeship (northern Poland) and in the Sudetic Mountains (southern Poland).

The ornithologists ringed the birds with the consent of the General Directorate of Environmental Protection, Poland (nos. 253/2012 and 259/2013).

Cloacal swabs from mallard ducks and black coots were obtained during the hunting season by two hunting associations in accordance with local hunting laws, special permission (with the consent of the Regional Directorate of Environmental Protection, Wrocław, Poland, no. WPN. 6205.67.2012.MK.1), and hunting programs. Samples from great cormorants were obtained during the annual population cull in Poland. All cloacal swabs from mallards, black coots, and great cormorants were collected in the lakes of the Lower Silesia region between August 15, 2012, and December 12, 2012. Cloacal swabs were collected from velvet scoters that were found dead in fishing nets on the Baltic coast in late winter and early spring.

The species of birds were grouped by their preferred habitats and/or behavior and were divided into waterfowl, songbirds, and birds kept in rescue centers, as well as migratory, partially migratory, or resident species (Table 1). The research was conducted with the consent of the 2^nd^ Local Ethical Committee for Animal Experiments (Wrocław, Poland; no. 41/2011).

### Bacterial isolation

All the samples were analyzed for *Salmonella* strains, which were isolated using the International Organization for Standardization Procedure PN-EN ISO 6579: 2003/A1: 2007. The samples were pre-enriched in nonselective buffered peptone water (Merck, Darmstadt, Germany) for 20 h at 37°C. After incubation, enriched modified semisolid Rappaport–Vassiliadis medium (Merck) was inoculated with the samples and incubated for 24 h at 41.5°C. The cultures were differentiated on solid xylose–lysine–deoxycholate agar (Merck) and on MacConkey agar (Merck), incubated for 24 h at 37°C. Three colonies per plate with the characteristics of *Salmonella* spp. were then spread onto nutrient agar (Merck) and incubated for 24 h at 37°C. The colonies were then identified biochemically with the API 20E system (Biomerieux, Marcy l’Etoile, France). All isolates were stored in Microbank vials (Microbank, Pro-Lab Diagnostics, Round Rock, TX, USA) at −70°C for further analysis.

### DNA extraction

After the cells were incubated overnight at 37°C on nutrient agar (Merck), the bacterial genomic DNA was extracted using the DNeasy® Blood & Tissue Kit (Qiagen, Valencia, CA, USA), according to the manufacturer’s instructions. The DNA was quantified spectrophotometrically (BioPhotometer, Eppendorf, Wesseling-Berzdorf, Germany) and stored at −20°C.

### *Salmonella* identification with PCR

The genus *Salmonella* was confirmed with multiplex PCR. *Salmonella* was identified at the genus level with the *invA* gene and at the subspecies level with the same multiplex PCR. The primer sequences used for amplification are summarized in Table 2. *Salmonella* was identified at the genus and subspecies levels according to Lee et al. [13].

**Table 2:** **Primers used in PCR to identify species and subspecies of** ***Salmonella*** **strains, according to Lee et al.** [13]

**Genes**	**Function of gene**	**Sequence of nucleotides**	**Size**
STM	encodes a putative inner membrane protein, specific for *S. enterica* subsp I	F-GGTGGCCTCGATGATTCCCG	137 bp
		R-CCCACTTGTAGCGAGCGCCG	
*stn*	encodes *Salmonella* enterotoxin and is specific for *S. enterica*	F-CGATCCCTTTCCCGCTATC	179 bp
		R-GGCGAATGAGACGCTTAAG	
*invA*	invasion protein, for simultaneous identification of *Salmonella* at the genus level	F-ACAGTGCTCGTTTACGACCTGAAT	244 bp
		R-AGACGACTGGTACTGATCGATAAT	
*gatD*	encodes the galacitol-1-phosphate dehydrogenase (gatD), contributes to acid production from galacitol	F-GGCGCCATTATTATCCTATTAC	501 bp
		R-CATTTCCCGGCTATTACAGGTAT	
*mdcA*	encodes the alpha subunit of the enzyme that contributes to malonate utilization	F-GGATGTACTCTTCCATCCCCAGT	728 bp
		R-CGTAGCGAGCATCTGGATATCTTT	
*fljB*	encodes phase 2 flagellin, enables differentiation between monophasic and diphasic subspecies	F-GACTCCATCCAGGCTGAAATCAC	848 bp
		R-CGGCTTTGCTGGCATTGTAG	

### *Salmonella* serotyping

*Salmonella* isolates were serotyped using single-factor antisera (Sifin, Berlin, Germany), according to the White–Kauffman–Le Minor scheme, focusing particularly on the five serovars mentioned above, which are monitored in poultry by the EU.

### Enterobacterial repetitive intergenic consensus (ERIC)-PCR

The genetic diversity of the isolated *Salmonella* ser. Typhimurium strains was analyzed with ERIC-PCR, using a protocol and primers (ERIC-R: 5′-ATGTAAGCTCCTGGGGATTCAC-3′; ERIC-F: 5′-AAGTAAGTGACTGGGGTGAGCG-3′) targeting the palindromic sequences of ERIC with the method described by Versalovic et al. [14].

### PCR detection of virulence genes

The virulence genotyping of *Salmonella* ser. Typhimurium (18 strains), *Salmonella* ser. Hadar (one strain), *Salmonella* ser*.* Virchow (one strain), and *Salmonella* ser*.* Infantis (one strain) was performed with the multiplex PCR described by Skyberg et al. [9]. The primers used in this experiment are listed in Table 3.

**Table 3:** **Primers used in PCR to detect the virulence genes in** ***Salmonella*** **strains, according to Skyberg et al.** [9]

**Genes**	**Function of gene**	**Sequence of nucleotides**	**Size**
*spvB*	Growth within host	F-CTATCAGCCCCGCACGGAGAGCAGTTTTTA	717 bp
		R-GGAGGAGGCGGTGGCGGTGGCATCATA	
*spiA*	Survival within macrophage	F-CCAGGGGTCGTTAGTGTATTGCGTGAGATG	550 bp
		R-CGCGTAACAAAGAACCCGTAGTGATGGATT	
*pagC*	Survival within macrophage	F-CGCCTTTTCCGTGGGGTATGC	454 bp
		R-GAAGCCGTTTATTTTTGTAGAGGAGATGTT	
*cdtB*	Host recognition/invasion	F-ACAACTGTCGCATCTCGCCCCGTCATT	268 bp
		R-CAATTTGCGTGGGTTCTGTAGGTGCGAGT	
*msgA*	Survival within macrophage	F-GCCAGGCGCACGCGAAATCATCC	189 bp
		R-GCGACCAGCCACATATCAGCCTCTTCAAAC	
*invA*	Host recognition/invasion	F-CTGGCGGTGGGTTTTGTTGTCTTCTCTATT	1070 bp
		R-AGTTTCTCCCCCTCTTCATGCGTTACCC	
*sipB*	Entry into nonphagocytic cells	F-GGACGCCGCCCGGGAAAAACTCTC	875 bp
		R-ACACTCCCGTCGCCGCCTTCACAA	
*prgH*	Host recognition/invasion	F-GCCCGAGCAGCCTGAGAAGTTAGAAA	756 bp
		R-TGAAATGAGCGCCCCTTGAGCCAGTC	
*span*	Entry into nonphagocytic cells	F-AAAAGCCGTGGAATCCGTTAGTGAAGT	504 bp
		R-CAGCGCTGGGGATTACCGTTTTG	
*orgA*	Host recognition/invasion	F-TTTTTGGCAATGCATCAGGGAACA	255 bp
		R-GGCGAAAGCGGGGACGGTATT	
*tolC*	Host recognition/invasion	F-TACCCAGGCGCAAAAAGAGGCTATC	161 bp
		R-CCGCGTTATCCAGGTTGTTGC	
*iron*	Iron acquisition	F-ACTGGCACGGCTCGCTGTCGCTCTAT	1205 bp
		R-CGCTTTACCGCCGTTCTGCCACTGC	
*sitC*	Iron acquisition	F-CAGTATATGCTCAACGCGATGTGGGTCTCC	768 bp
		R-CGGGGCGAAAATAAAGGCTGTGATGAAC	
*lpfC*	Host recognition/invasion	F-GCCCCGCCTGAAGCCTGTGTTGC	641 bp
		R-AGGTCGCCGCTGTTTGAGGTTGGATA	
*sifA*	Filamentous structure formation	F-TTTGCCGAACGCGCCCCCACACG	449 bp
		R-GTTGCCTTTTCTTGCGCTTTCCACCCATCT	
*sopB*	Host recognition/invasion	F-CGGACCGGCCAGCAACAAAACAAGAAGAAG	220 bp
		R-TAGTGATGCCCGTTATGCGTGAGTGTATT	
*pefA*	Host recognition/invasion	F-GCGCCGCTCAGCCGAACCAG	157 bp
		R-GCAGCAGAAGCCCAGGAAACAGTG	

### Positive controls

Two strains, *Salmonella* ser*.* Typhimurium (ATCC # 14028) and *Salmonella* ser*.* Hadar (laboratory strain), previously shown to contain all the genes tested (*Salmonella* species, subspecies and virulence genes), served as positive control strains. Identity of *Salmonella* ser*.* Hadar strain was verified by sequencing.

## Results

### Isolation and identification

*Salmonella* species were isolated from 64 (6.4%) of the 1000 samples collected (Tables 1 and 4). Most of the positive samples came from the Eurasian siskin (*Carduelis spinus*) (16/48, 33.33%) and the greenfinch (*Carduelis chloris*; 10/30, 33.33%). Positive samples were also collected from 13 other species, including the great cormorant (*Phalacrocorax carbo*; 8/77, 10.39%), great tit (*Parus major*; 10/109, 9.17%), and mallard duck (*Anas platyrhynchos*; 8/121, 6.61%). A positive sample was also obtained from a Eurasian marsh harrier (*Circus aeruginosus*; 1/1, 100.00%). This last sample was collected from the bird during its second day at a wildlife rescue center in Lower Silesia before antibiotic treatment was commenced (Table 1).

**Table 4:** **Species, subspecies, and serotypes of** ***Salmonella*** **isolates collected**

**Species**	**Subspecies**	**Serotype**	**Origin**	**Number of isolates**
*Salmonella enterica*	*enterica* (I)	Typhimurium 4,12:i:1,2	Eurasian siskin	7
			Greenfinch	3
			Mallard duck	3
			Redpoll	1
			Common wood pigeon	1
			Blue tit	1
			Great tit	1
			Blackbird	1
		Infantis 6,7:r:1,5	Common starling	1
		Virchow 6,7:r:1,2	Common starling	1
		Hadar 6,8:z_10_:e,n,x	Mallard duck	1
		others	Eurasian siskin	8
			Great cormorant	7
			Mallard duck	4
			Common starling	1
			Greenfinch	7
			Great tit	1
			Rook	1
			Eurasian marsh harrier	1
			Eurasian tree sparrow	1
	salamae (II)	others	Eurasian tree sparrow	1
			Great cormorant	1
			Great tit	8
			Common swift	1
	houtenae (IV)	others	Mallard duck	1

The collected *Salmonella* strains all belonged to one of three subspecies: *enterica* (81.25%), *salamae* (17.19%), or *houtenae* (1.56%). *S. enterica* subsp*. enterica* was isolated from the vast majority of bird species, but *S. enterica* subsp. *salamae* was collected from four species of birds (Eurasian tree sparrow, great cormorant, great tit, and common swift). Only one strain, isolated from a mallard duck, was *S. enterica* subsp. *houtenae* (Table 4).

Among the *Salmonella* strains collected, four of the five serovars of *Salmonella* that are constantly monitored by the EU in poultry were found in free-living birds. Eighteen strains belonged to ser. Typhimurium (28.13%), one to ser. Infantis (1.56%), one to ser. Virchow (1.56%), and one to ser. Hadar (1.56%). No *Salmonella* ser*.* Enteritidis was isolated from any sample collected from free-living birds. Serovars Virchow and Infantis were isolated from two very young starlings. Serovar Hadar was isolated from the mallard duck. Serovar Typhimurium was the serovar isolated from the greatest number of bird species (Table 4).

**Figure 1:**
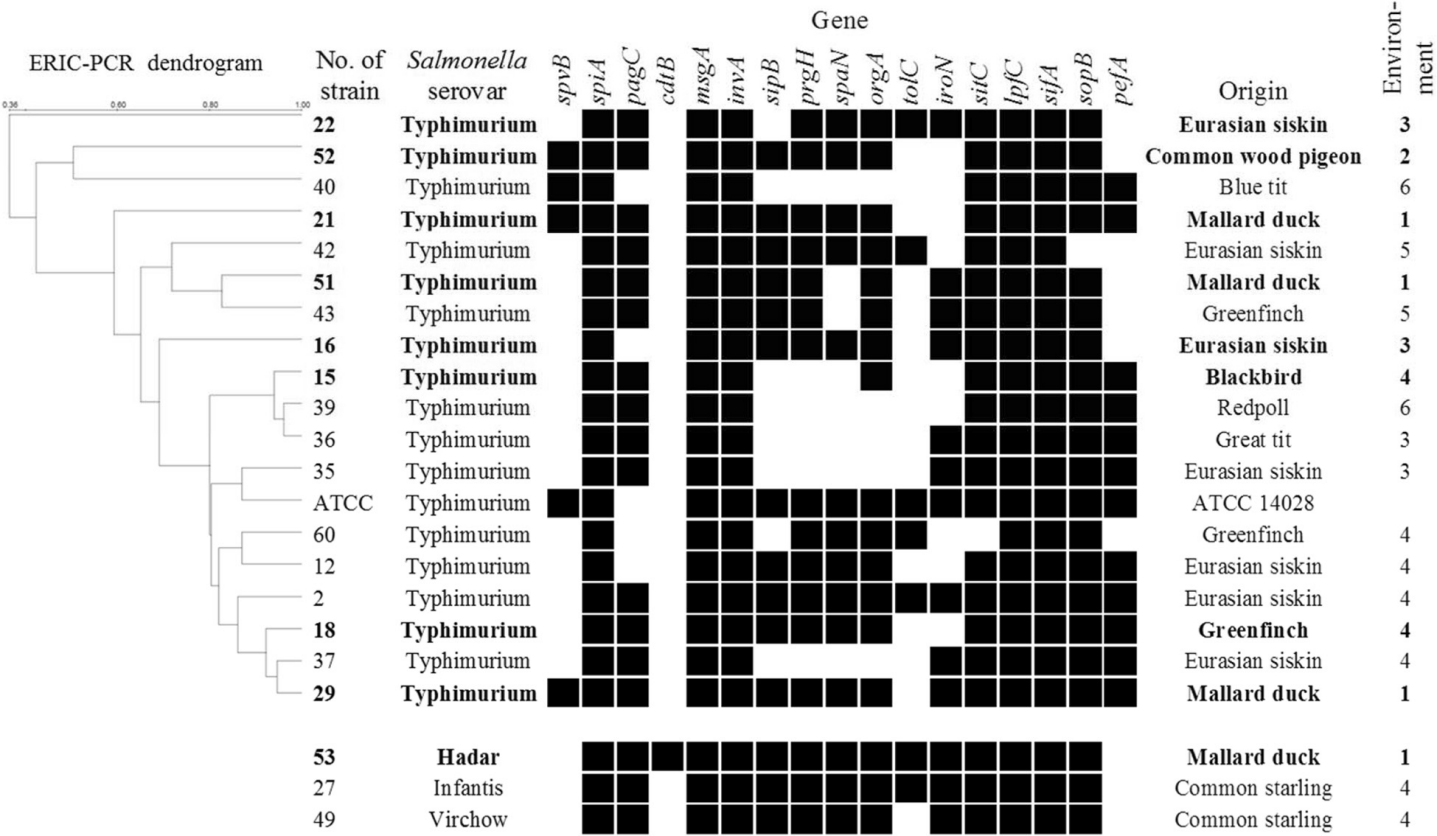
**The ERIC-PCR analysis and virulence genes of** ***Salmonella*** **serovars: Typhimurium, Hadar, Infantis, and Virchow.** Black indicates the presence of the gene, white indicates the absence of the gene, boldfaces in text indicate that the strain was isolated from dead bird; explanation of environmental numbers, see legend of Table 1.

ERIC-PCR categorized the 18 *Salmonella* ser. Typhimurium strains obtained from free-living birds into different profiles. One strain remained as nonhomologous to any other strain. The *Salmonella* ser. Typhimurium strains showed no correlation with bird species (e.g., isolates from Eurasian siskin nos. 22, 42, and 16 differed), but similarity was observed among the strains isolated from the same environmental areas (strain nos. 60, 12, 2, 18, and 37 were similar). The first cluster included strains collected in two regions: Wrocław city center and suburbs. The *Salmonella* ser. Typhimurium isolates collected from dead birds also displayed genetic diversity (Figure 1).

All the isolated *Salmonella* ser. Typhimurium strains contained the *spiA*, *msg*A, *inv*A, *lpf*C, and *sif*A genes; 94.45% isolates also contained the *sit*C and *sop*B genes. None of the *Salmonella* ser. Typhimurium strains contained the *cdtB* gene. The presence of other genes was investigated*.* The genes in the *Salmonella* ser. Typhimurium strains were highly variable. The one *Salmonella* ser. Hadar strain contained all the tested genes, except *spv*B and *pef*A; the one *Salmonella* ser. Infantis strain contained all the tested genes, except *spv*B, *pef*A, and *cdtB*; and *the one Salmonella* ser. Virchow contained all the tested genes, except *spv*B, *pef*A, *cdtB*, and *tolC*. The prevalence of virulence genes in the *Salmonella* ser. Typhimurium strains varied among the live and dead free-living birds (Figure 1).

## Discussion

The results of this study confirm that *Salmonella* ser. Typhimurium, one of the most frequently reported serotypes in human salmonellosis in the EU, occurs among free-living birds. Three other serotypes monitored in poultry flocks by the EU, Hadar, Virchow, and Infantis, were also present among the free-living bird populations. Free-living birds are considered to be potential carriers of these bacteria and to play a role in the ecology and circulation of several zoonotic pathogens [4-7].

In Central Europe, only a few reports of salmonellosis in wild birds have been published, in the 1990s [7,15]. In Poland, all similar research has been conducted in the small northern region of the country, and there is a dearth of wide epidemiological studies in this field [16,17].

*Salmonella* infection may occur as a visible illness or be asymptomatic, depending upon the bird species. It may also result from exposure to an environment that has been contaminated by infected humans or livestock [15,18,19]. Migratory birds, in particular, are potential reservoirs for bacterial agents [20]. Many wild passerines have been documented as carriers of *Salmonella* strains, and their involvement in the transmission of *Salmonella* to mammals and people has been suggested [21,22]. In this study, most of the positive samples came from garden bird species: Eurasian siskins and greenfinches. These results are compatible with the findings of Hughes et al. [23], who reported that *Salmonella* caused mortality in wild birds, particularly garden birds, in the United Kingdom. Lawson et al. [24] also reported that house sparrows and greenfinches are particularly susceptible to salmonellosis. Consistent with our results, it has also been documented that the *Salmonella* serovar most commonly isolated from free-living birds is ser. Typhimurium, which appears to be adapted to some avian species that frequent bird feeders, including songbirds [25]. The results of the present study clearly show that the bird species with the highest proportion of *Salmonella*-positive samples also frequented bird feeders. Both European siskins and greenfinches seem to be particularly susceptible to *Salmonella* ser. Typhimurium. This result suggests a high incidence of *Salmonella* exposure near bird feeders during winter and its transmission to birds. It can be inferred that the risk of transmission from the feces of infected wild passerines to uninfected birds is high, especially in urban areas with many bird feeders. As reported by Hamer et al. [25] and later noted by Borreli et al. [26], the key features of the urban environment that promote the transmission of pathogens include increased host contact rates, susceptibility to infection, high rates of pathogen introduction, pollution and stress (which reduce the host immune function), and warmer microclimates with reduced seasonality (which allow the environmental persistence of some pathogens). These factors may explain the increased frequency of salmonellosis we observed in birds between February and April during a prolonged winter in Poland in 2013 (data not shown in the table). In the United Kingdom, Hughes et al. [23] reported similar peaks of *Salmonella* isolation in January and February. Kapperud et al. [18] documented the seasonality of salmonellosis outbreaks, simultaneously in people and wild passerines, in Norway in 1998, which appeared in both groups between January and April. It is also possible that salmonellosis outbreaks in free-living birds during this time of year are associated with the feeding of birds by people. Supplemental feeding creates high densities of birds, high concentrations of feces, and stress arising from social interactions, which may also increase the prevalence of some bacterial species among wild birds [25]. It has been suggested that certain strains of *Salmonella* ser. Typhimurium are associated with different groups of wild birds [19,23,27-30]. This is supported by the recovery of this serotype from mallard ducks and great cormorants in this study.

Daoust and Prescott [31] reported that salmonellosis can cause sporadic mortality, particularly among birds around feeders, but also in young birds in large breeding colonies. These results prompted us to check the prevalence of selected virulence genes (encoding virulence factors) that are also capable of causing human infections [9,10,12]. In this study, we have demonstrated the great variability in the virulence genes present in isolated *Salmonella* strains in both dead and live birds, and among birds of the same species.

Similar results for the prevalence of virulence genes have been reported by other researchers. Skyberg et al. [9] recorded that the same 17 virulence genes were widespread in many *Salmonella* serovars isolated from both sick and healthy birds. Similar findings were recorded by Mezal et al. [11] among environmental samples, including dust, water, and other materials from poultry houses. Our study confirms the presence of the same virulence genes, which might play important roles in the bacterial invasion and survival in the host of *Salmonella* isolates collected from different species of free-living birds, as in human clinical isolates. These findings suggest that like poultry flocks, poultry houses, and the environments around poultry farms, wild birds might be a source of *Salmonella* strains that are pathogenic to people. We also found evidence that the genetic homogeneity of some *Salmonella* serovars (e.g., ser. Typhimurium) is changeable, but is greater among different species of birds that spend their lives in similar geographical localities. Chrząstek et al. [32] also demonstrated a correlation between genetic homogeneity and the geographical origin of the host, but with *Pasteurella multocida* strains collected from poultry in different regions in Poland. Our results confirm the genetic similarity of *Salmonella* ser. Typhimurium strains isolated from wild birds in the area of Wrocław.

## Conclusions

*Salmonella* species are present in populations of free-living bird species, especially in birds sampled in urbanized areas. Some wild avian species are reservoirs for *Salmonella* serotypes, especially *Salmonella* ser. Typhimurium Most of the positive samples came from the Eurasian siskin and the greenfinch. The *Salmonella* isolates presented the same virulence genes as in human clinical isolates. This suggests a potential risk for people feeding infected wild birds.

### Availability of supporting data

The study was conducted with the special consent mentioned in the text above (see Methods). All dead birds (except game birds) were found already dead and brought to the clinic. Game birds were hunted and collected by hunters in accordance with local hunting laws. Samples of great cormorants were obtained during the annual population cull in Poland, in accordance with the annual specifications of the Regional Directorate of Environmental Protection.
